# Analysis of the role of intratecal liposomal cytarabine in the prophylaxis and treatment of central nervous system lymphomatosis: The Balearic Lymphoma Group experience

**DOI:** 10.1371/journal.pone.0179595

**Published:** 2017-06-30

**Authors:** Marta García-Recio, Antonia Cladera, Leyre Bento, Julia Dominguez, Silvia Ruiz de Gracia, Francesca Sartori, Raquel Del Campo, Lucia García, Carmen Ballester, Jordi Gines, Joan Bargay, Antonia Sampol, Antonio Gutiérrez

**Affiliations:** 1 Hematology department, Son Espases University Hospital, Palma de Mallorca, Balearic Islands, Spain; 2 Hematology department, Hospital Son Llatzer, Palma de Mallorca, Balearic Islands, Spain; 3 Pharmacy department, Son Espases University Hospital, Palma de Mallorca, Balearic Islands, Spain; Instituto Cajal-CSIC, SPAIN

## Abstract

Central nervous system (CNS) lymphomatosis is a fatal complication of aggressive non-Hodgkin lymphoma (NHL). In lymphoblastic or Burkitt lymphoma, without specific CNS prophylaxis the risk of CNS relapse is 20–30%. DLBCL has a lower risk of relapse (around 5%) but several factors increase its incidence. There is no consensus or trials to conclude which is the best CNS prophylaxis. Best results seem to be associated with the use of intravenous (iv) high-dose methotrexate (HDMTX) but with a significant toxicity. Other options are the administration of intrathecal (IT) MTX, cytarabine or liposomal cytarabine (ITLC). Our aim is to analyze the experience of the centers of the Balearic Lymphoma Group (BLG) about the toxicity and efficacy of ITLC in the prophylaxis and therapy of CNS lymphomatosis. We retrospectively reviewed cases from 2005 to 2015 (n = 58) treated with ITLC. Our toxicity results were: 33% headache, 20% neurological deficits, 11% nausea, 9% dizziness, 4% vomiting, 4% fever, 2% transient blindness and 2% photophobia. In the prophylactic cohort (n = 26) with a median follow-up of 55 months (17–81) only 3 CNS relapses (11%) were observed (testicular DLBCL, Burkitt and plasmablastic lymphoma, with a cumulative incidence of 8%, 14% and 20% respectively). In the treatment cohort (n = 32), CSF complete clearance was obtained in 77% cases. Median OS was 6 months (0–16). Death causes were lymphoma progression (19 patients, 79%), treatment toxicity (2 patients) and non-related (3 patients, 12%). Toxicity profile was good especially when concomitant dexamethasone was administered. In the prophylactic cohort the incidence of CNS relapse in DLBCL group was similar to previously reported for HDMTX and much better than IT MTX. A high number of ITLC injections was associated with better rates of CSF clearance, clinical responses, PFS and lower relapses. Survival is still poor in CNS lymphomatosis and new therapeutic approaches are still needed.

## Introduction

The incidence of CNS involvement in non Hodgkin lymphoma (NHL) varies among different histological subtypes (<3% for indolent lymphomas and up to 30% for highly-aggressive lymphomas). CNS affection may be detected at diagnosis as a malignancy exclusively located in CNS (primary CNS lymphomas) or in association with systemic disease. Also it may be a site of relapse from a systemic lymphoma [1]. CNS relapse may involve the brain parenchyma, spinal cord, leptomeninges or eye.

Diffuse large B-cell lymphoma (DLBCL) is the most common NHL. The risk of CNS involvement in DLBCL treated with CHOP-like regimens is only 5% but confers a very poor prognosis (median survival of 4–5 months). It is currently assumed that CNS relapse in NHL is likely due to occult malignant cells which are present but have not been detected in the CNS at diagnosis [2].

As the risk of CNS relapse in DLBCL is low it is controversial when to administer CNS prophylaxis[3]. For this reason different groups focused on defining risk factors that could identify those cases candidates to prophylaxis of CNS involvement[4,5]. Boehme et al.[6] first defined raised lactate dehydrogenase (LDH), more than 1 extranodal site of disease and presence of B symptoms as independent predictors of CNS relapse risk. Later on, specific anatomical affections, such as testicular, breast, epidural space or cranial sinuses, were included in these risk factors guidelines[7].

Another important and controversial question is which the best way to administer this CNS prophylaxis is. Some of the best results have been obtained with systemic chemotherapy which crosses the blood-brain barrier (BBB) such as intravenous (IV) high dose methotrexate (HDMTX) that results in a therapeutic serum level higher than IT bolus administration[8], a typical way of administering CNS prophylaxis. Despite this, ongoing research is trying to improve IT therapies that avoid the inconveniences of HDMTX (toxicity, hospital admission…). One of these promising IT drugs is liposomal cytarabine (ITLC) (DepoCyte), that sustains the therapeutic concentration for at least 14 days.

In this manuscript we review our experience with ITLC used in both the treatment and the prophylaxis setting in the centres of the Balearic Group of Lymphoma both as prophylaxis or treatment of the meningeal lymphomatosis.

## Materials and methods

### Patient and study design

We present a retrospective multicentric study carried out inside the Balearic Lymphoma Group in Son Espases University Hospital and Son Llatzer Hospital of Palma de Mallorca. To avoid selection bias we obtained the patients’ data from the Pharmacy and Pathology Departments registries. From June 2005 to June 2015, 58 patients with lymphoma were treated with ITCL as prophylaxis (n = 26) in high risk cases or treatment (n = 32) of meningeal lymphomatosis and were included in the present study. Standard significant clinical data was retrospectively obtained from the records including diagnosis, staging, concomitant induction treatment received and CSF involvement. Eight-colours flow cytometry (FC) was used to assess CSF involvement at diagnosis and at the evaluation of response or follow-up. CNS involvement was assessed also through neurological examination and patients with signs or symptoms of CNS involvement underwent imaging studies (CT or MRI).

### Response, toxicity assessment and follow-up criteria

We used the standard guidelines for evaluations[9] including physical examination, complete blood counts, serum biochemistry, bone marrow aspiration or biopsy, and radiological studies. We used the version 4.0 of the Common Terminology Criteria for Adverse Events of the National Cancer Institute (NCI-CTCAE v4.0) criteria to assess the toxicity associated to the treatment[10]. We always considered toxic mortality if it was related to the procedure.

The retrospective study was approved by the local ethics committee: Comitè etic de d’Investigació clínica de les Illes Balears (CEIB-IB) with the number IB 1680/11PI. Written inform consent were obtained from alive patients.

### Statistical methods

PFS (time to progression/relapse) and overall survival (OS) (time to death) were measured from the date of first ITLC administration and were estimated according to the Kaplan-Meier method[11] We performed the comparisons between those interest variables with the log-rank test[12]. Comparison between categorical variables was made with the Chi-square of Fisher exact test as appropriate. All reported p-values were two-sided and statistical significance was defined at p <0.05.

## Results

### Characteristics of patients

We show in S1 Table main characteristics of patients, also comparing both treatment cohorts: prophylaxis (n = 26) and treatment (n = 32). In both groups there was a clear male prevalence (3:1) and a median age of 53 years (10–85). The most common histologic diagnosis was DLBCL (45%) followed by lymphoblastic leukemia/lymphoma or Burkitt lymphoma (BL) (26%). The majority of patients were diagnosed in stage IV (78%).

Most patients in the prophylaxis cohort received CHOP-like conventional chemotherapy while in the treatment cohort most cases were treated with intensive CNS or acute leukemia-type schemes (HyperCVAD/AM, ALL or BL intensive protocols from Spanish cooperative groups such as PETHEMA or SEHOP). In most cases, ITLC was accompanied by prophylactic IT and/or oral dexamethasone in order to avoid chemical arachnoiditis.

### Toxicity analysis

The total number of ITLC injections included in our study was 180: 72 in the 26 patients of the prophylactic cohort and 108 in the 32 patients of the therapeutic cohort. Overall adverse effect (AE) incidence was of 34%, most grades 1–2. As shown in S2 Table we found no statistically significant differences in AE between both cohorts. Headache was the most frequent side effect both in the prophylaxis (29%) and the treatment cohort (35%) with a global incidence of 33%. Neurological deficits (vestibular syndrome or radiculopathy), nausea or dizziness appear respectively in 20% (17% vs 23%), 11% (8% vs 13%) and 9% (12% vs 6%). One case in every cohort presented transient blindness grade 3 after ITLC administration. Photophobia presented in one treatment case (3%) and fever in one case in the prophylaxis (4%) and another in the treatment (3%) cohort.

Most patients in the prophylaxis and treatment cohorts (respectively, 77% and 69%) received IT prophylaxis with dexamethasone concomitant to ITLC administration. We found no differences in the incidence of AE in patients receiving or not receiving IT dexamethasone prophylaxis. However, most patients not receiving IT dexamethasone received oral dexamethasone. Of note, both cases that experienced transient blindness after ITLC had received dexamethasone IT prophylaxis.

### Role of liposomal cytarabine in the prophylaxis of CNS lymphomatosis

In the prophylactic cohort (n = 26) the median number of administered liposomal cytarabine injections was 2.5 (1–7). As shown in S1 Table, most patients were high risk DLBCL (46%), Burkitt or lymphoblastic lymphoma / leukemia (27%) or plasmablastic lymphoma (19%). All DLBCL or plasmablastic lymphomas received concomitant standard R-CHOP-like therapy while BL/lymphoblastic cases were induced with intensive lymphoblastic-like polychemotherapy. All DLBCL patients had a high risk of CNS relapse in terms of having a high risk location (testicular, breast, epidural space of cranial sinuses) or at least two of the following features: IPI 3–5, elevated LDH and ≥ 2 extranodal localizations[7].

With a median follow-up for alive patients of 55 months (17–81) only 3 CNS relapses (11%) were observed in a testicular DLBCL, Burkitt and plasmablastic lymphoma. This represents a cumulative incidence of CNS relapse of 8%, 14% and 20%, respectively for DLBCL, BL/lymphoblastic cases or plasmablastic lymphoma.

At last follow-up, 15 (58%) patients were alive: 9 (75%) in the DLBCL, 2 (29%) BL/lymphoblastic group and 2 (40%) plasmablastic lymphoma. Causes of death were progression of lymphoma in 10 cases (91%) and sepsis in 1 (9%). Median OS for the whole series was not reached with an actuarial 4 year-OS of 73%, 29% and 40%, respectively for DLBCL, BL/lymphoblastic cases and plasmablastic lymphoma (the last two groups of patients with a median OS of 10 and 13.7 months, respectively). Median PFS was 72 months (0–165) with 12 (46%) patients relapsing: 4 (33%), 5 (71%) and 3 (60%), respectively for DLBCL, BL/lymphoblastic cases and plasmablastic lymphoma.

### Efficacy of liposomal cytarabine in the treatment of CNS lymphomatosis

In the therapeutic cohort (n = 32) the median number of liposomal cytarabine injections was 3 (1–8). Characteristics of patients are shown in S1 Table. Response to therapy was evaluated in terms of neurologic improvement as well as CSF clearance (S3 Table). Around 50% of patients obtained a neurologic CR with ITLC, being this CR rate higher in patients with PCNSL (80%). Complete clearance of CSF by high-resolution immunophenotyping was achieved in 78% of patients: 100%, 86% and 75% of patients with PCNSL, DLBCL and BL/lymphoblastic lymphoma, respectively. Rates of CSF clearance (p = 0.042) and clinical response (p = 0.036) were significantly higher in those patients receiving more ITLC injections. The apparent low number of median ITLC injections was justified by the high rate of patients that progressed during therapy (59%). Only 3 patients (6%) discontinued ITLC due to the development of side effects (ej. neurological deficits or dizziness) while most ended combined therapy including HDMTX, CNS radiotherapy or SCT (31%). Interestingly only one of these last patients suffered a late CNS progression at last follow up. Another patient died due to an unrelated cause (suicide).

Median PFS was 7 months (5–8) and actuarial PFS at 1 year was 36% with 16 patients (50%) relapsing in CNS and other 4 (12%) relapsing outside of CNS with no differences regarding diagnosis or induction treatment received. Higher number of ITLC injections was associated with a lower incidence of CNS relapse (p = 0.048). In fact, 12y-PFS was significantly better for patients receiving 4 or more ITLC injections: 69% versus 33% for patients treated with 3 or less ITLC injections (p = 0.031). Median OS was 6 months (0–16). Causes of death were lymphoma progression in 19 patients (79%), treatment toxicity in 4 (17%) (infections) and other non-related in 1 (4%) (suicide).

## Discussion

CNS relapse is a generally fatal complication of aggressive NHL. Without specific CNS prophylaxis the risk of CNS relapse is higher (up to 30%)[1] in patients with very aggressive NHL such as Burkitt or lymphoblastic lymphoma/leukemia. DLBCL and other aggressive lymphomas (primary mediastinal or peripheral T-cell lymphoma) have an overall lower risk (around 5%) but several factors have been identified that increase the incidence of CNS relapse to levels similar to very aggressive NHL: high LDH and more than 1 extranodal site, as well as several high risk lymphoma locations such as testicular[6,13].

In all these high risk cases the administration of a CNS prophylaxis is recommended by guidelines. However there is no consensus regarding the best type of prophylaxis. Different therapeutic modalities have been used: radiation therapy, systemic chemotherapy, IT or a combination. Radiation therapy, although effective, often associates unacceptable late adverse effects (secondary neoplasms, endocrinopathy, neurocognitive dysfunction and neurotoxicity) [14].

For this reason, systemic intravenous chemotherapy able to cross BBB and IT combinations have been tested. However, high-dose cytarabine is associated with liver and cerebellar dysfunction, mucositis, diarrhea, rash and fever, and HDMTX, associated with renal dysfunction, transient hepatitis, mucositis and occasionally neurotoxicity[15]. Moreover it is difficult to maintain prolonged therapeutic drug concentrations in CSF when administered intravenously and their administration may delay standard chemotherapy[16].

IT prophylaxis include administration of HDMTX alone or with cytarabine (triple IT), rituximab or liposomal cytarabine. Conventional IT cytarabine half-life is 4.5 hours after IT administration vs 100 to 263 hours of liposomal formulation of cytarabine [16]. IT MTX half-life is of 3.4 hours thus requiring 2–3 administrations per week depending on regimen. ITLC is a slow-release formulation of cytarabine designed to ensure prolonged drug exposure when administered.

There are no randomized clinical trials comparing the efficacy and toxicity of the above mentioned types of CNS prophylaxis. However, a recent retrospective study performed in high risk DLBCL compared the three types of CNS prophylaxis showing a much lower incidence of relapse in CNS in those cases receiving intensive regimens including antimetabolites (HyperCVAD/AM or CODOXM/IVAC) (2.3%) or R-CHOP with IT MTX and 2 cycles of IV HDMTX (8%) compared to patients receiving only IT MTX with a 24% CNS relapse. Although this information is retrospective and needs confirmation in a randomized clinical trial, it seems that CNS prophylaxis with IV HDMTX or cytarabine may be associated to a lower incidence of CNS relapse when compared to IT MTX alone[4]. Good results have been reported in BL and unclassifiable lymphomas with features intermediate between BL and DLBCL, in a prospective study (n = 30) with a recurrence rate of 3.4% by modifying CODOX-M/IVAC regimen (adding rituximab and LC) [17].

Several groups reported the use of ITLC in the prophylaxis and treatment of CNS involvement in high risk subgroups of aggressive lymphoma. Kumiega B et al. [18] reported a series of 19 patients treated with ITLC prophylaxis. In their experience only one patient (5.5%) had CNS relapse after this prophylaxis. Two years later, the Polish lymphoma research group presented a series of 120 adult patients in high risk of CNS involvement (n = 95) and patients with CNS lymphomatosis (n = 25)[19]. None of the prophylactic patients (0%) experienced CNS relapse. ORR of patients included in treatment cohort was 76%. González-Barca E. et al [20] reported a large series of 129 patients diagnosed with DLBCL who received a first line of systemic therapy combined with ITLC obtaining low rates of toxicity and no CNS relapse after 40.1 months of follow-up.

A recent randomized trial comparing ITT versus ITLC CNS prophylaxis in ALL patients showed a 6% CNS relapses with ITT prophylaxis vs 3% using ITLC prophylaxis [21]. Different studies proved conventional IT prophylaxis therapy (ITT, Rituximab or MTX) to be insufficient avoiding CNS relapses [6,13,22]. So far, IV HDMTX prophylaxis has the best results avoiding CNS relapses (around 3–8% in different series)[4,23].

In our study we present a series of 26 patients that received ITLC as CNS prophylaxis. Considering our high risk DLBCL patients we observed an 8% of relapses that it is similar to the previously reported for patients receiving systemic HDMTX and much better that those reported using IT MTX. Although this is a small and retrospective series from which it is not possible to draw definitive conclusions, it could justify the development of a randomized clinical trial to test the hypothesis that ITLC prophylaxis could achieve similar good results as HDMTX with less toxicity.

In the rituximab era, most CNS relapses occur in the brain parenchyma and this could generate the concern that ITLC could not prevent parenchymal relapses. However, in our prophylaxis cohort we only had 2 of 3 CNS relapses involving brain parenchyma and no one exclusively as they also suffered leptomeningeal or systemic progression. Similarly, most series reported with ITLC prophylaxis did not communicate a higher number of parenchymal CNS relapses. So, prospective trials should search this point that it seems a logical concern related to ITLC instead of HD MTX prophylaxis.

Our results in terms of toxicity seem better than previously reported. In fact, in our series we observed only a 34% of AEs, mostly grade 1–2. This compares favourably to other recent series that present a 60% of AEs without dexamethasone arm vs 44.4% in dexamethasone arm[21]. Other studies also reported a high rate of AEs (63.1% to 79.2%) after ITLC administration[18,19,24]. This lower rate of AEs presented in our study may be explained by the routine intravenous or oral dexamethasone administration in our series.

On the other hand, the results obtained using ITLC in the treatment of meningeal lymphomatosis were good in terms of CSF clearance (75% to 100% depending on histology type). However, the main problem was related to the high number of early relapses (59%), most of them in the CNS that led to poor median PFS and OS: 7 and 6 months respectively. Although it is not clear the best number of ITLC injections in these cases, in our patients the rates of CSF clearance and clinical response were significantly higher in those patients receiving more ITLC injections. However, in our series most ITLC discontinuations were related to disease progression and only 1 patient that ended programmed combined ITLC therapy including HDMTX, CNS radiotherapy or SCT suffered a late CNS relapse.

Recent reports studied the role of ITLC in meningeal lymphomatosis presenting response rates of 85.7% with concomitant CNS-penetrating chemotherapy vs 83.3% without concomitant CNS-penetrating chemotherapy and a median event-free survival of 10 months[19]. Other groups reported similar response rates (81.8%) in this subgroup of CNS-affected patients and an OS of 11 months[25]. All this highlights the poor prognosis of meningeal lymphomatosis in which new drugs or therapeutic approaches are needed to improve results.

Finally, we conclude toxicity profile of ITLC is good especially when concomitant IT and oral dexamethasone is administered. In the prophylactic cohort, the incidence of CNS relapse was low in the high-risk DLBCL group similar to previously reported for systemic HDMTX and much better than IT MTX. ITLC may be a good alternative for CNS prophylaxis but it should be proved in randomized clinical trials. Patients in the treatment cohort had a high rate of clearance of CSF but survival is still poor in CNS lymphomatosis and new drugs or therapeutic approaches are still needed. Higher number of ITLC injections was associated with better rates of CSF clearance and clinical responses as well as better CNS PFS and lower relapses in CNS.

## Supporting information

S1 Table Patient characteristics. DLBCL: diffuse large B-cell lymphoma, CNS: central nervous system, CHOP: cyclophosphamide, vincristine, doxorrubicine and prednisone.
(DOCX)

S2 Table Toxicity. (DOCX)

S3 Table Response evaluation in neuro-meningeal lymphomatosis. DLBCL: diffuse large B-cell lymphoma, BL: Burkitt lymphoma, PCNSL: primary central nervous system lymphoma, CR: complete response, PR: partial response, SD: stable disease, PD: progressive disease, CSF: cerebrospinal fluid.
(DOCX)
