# Supplementary material for: Analysis of the role of intratecal liposomal cytarabine in the prophylaxis and treatment of central nervous system lymphomatosis: The Balearic Lymphoma Group experience
Source: PLoS One. 2017 Jun 30;12(6):e0179595. doi: 10.1371/journal.pone.0179595 (PMC5493300; doi:10.1371/journal.pone.0179595)
Supplement: S1 Table — DLBCL: diffuse large B-cell lymphoma, CNS: central nervous system, CHOP: cyclophosphamide, vincristine, doxorrubicine and prednisone. (DOCX) [file pone.0179595.s001.docx]

**Supporting information**

**S1 Table. Patient characteristics**

| Characteristics | Global group  (n=58) | Prophylaxis cohort (n=26) | Treatment cohort  (n=32) |
| --- | --- | --- | --- |
| Median age (range) | 53 (10-85) | 47 (12-85) | 55 (10-81) |
| Gender:   - Male - Female | 44 (76%)  14 (24%) | 19 (73%)  7 (27%) | 25 (78%)  7 (22%) |
| Diagnostic:   - DLBCL - Lymphoblastic Lymphoma / Leukemia / Burkitt lymphoma - Plasmablastic lymphoma - Primary CNS Lymphoma - Mantle Cell Lymphoma - Peripheral T-cell lymphoma - Folicular Lymphoma | 26 (45%)  15 (26%)  5 (9%)  6 (10%)  3 (5%)  2 (3%)  1 (2%) | 12 (46%)  7 (27%)  5 (19%)  1 (4%)  1 (4%)  0 (0%)  0 (0%) | 14 (44%)  8 (25%)  0 (0%)  5 (16%)  2 (6%)  2 (6%)  1 (3%) |
| Stage at diagnosis:   - I - II - III - IV | 3 (5%)  6 (10%)  4 (7%)  45 (78%) | 0 (0%)  4 (15%)  2 (8%)  20 (77%) | 3 (9%)  2 (6%)  2 (6%)  25 (78%) |
| First line treatment:   - Intensive treatment acute leukemia-*like* - Intensive treatment for CNS - Conventional chemotherapy CHOP-*like* - Other | 14 (24%)  11 (19%)  23 (40%)  10 (17%) | 7 (27%)  1 (4%)  17 (65%)  1 (4%) | 7 (22%)  10 (31%)  6 (19%)  9 (28%) |

DLBCL: diffuse large B-cell lymphoma, CNS: central nervous system, CHOP: cyclophosphamide, vincristine, doxorrubicine and prednisone.
