# Supplementary material for: Analysis of the role of intratecal liposomal cytarabine in the prophylaxis and treatment of central nervous system lymphomatosis: The Balearic Lymphoma Group experience
Source: PLoS One. 2017 Jun 30;12(6):e0179595. doi: 10.1371/journal.pone.0179595 (PMC5493300; doi:10.1371/journal.pone.0179595)
Supplement: S2 Table — (DOCX) [file pone.0179595.s002.docx]

**Supporting information**

**S2 Table. Toxicity**

| **Adverse effects: N (%)** | **Global group**  **(n=58)** | **Prophylaxis cohort (n=26)** | **Treatment cohort**  **(n=32)** | **P** |
| --- | --- | --- | --- | --- |
| **Nausea** | 6 (11%) | 2 (8%) | 4 (13%) | 0.69 |
| **Vomiting** | 2 (4%) | 0 (0%) | 2 (6%) | 0.5 |
| **Headache** | 18 (33%) | 7 (29%) | 11 (35%) | 0.77 |
| **Fever** | 2 (4%) | 1 (4%) | 1 (3%) | 1 |
| **Neurological deficits** | 11 (20%) | 4 (17%) | 7 (23%) | 0.74 |
| **Dizziness** | 5 (9%) | 3 (12%) | 2 (6%) | 0.64 |
| **Blindness** | 2 (4%) | 1 (4%) | 1 (3%) | 1 |
| **Photophobia** | 1 (2%) | 0 (0%) | 1 (3%) | 1 |
