# Supplementary material for: Analysis of the role of intratecal liposomal cytarabine in the prophylaxis and treatment of central nervous system lymphomatosis: The Balearic Lymphoma Group experience
Source: PLoS One. 2017 Jun 30;12(6):e0179595. doi: 10.1371/journal.pone.0179595 (PMC5493300; doi:10.1371/journal.pone.0179595)
Supplement: S3 Table — DLBCL: diffuse large B-cell lymphoma, BL: Burkitt lymphoma, PCNSL: primary central nervous system lymphoma, CR: complete response, PR: partial response, SD: stable disease, PD: progressive disease, CSF: cerebrospinal fluid. (DOCX) [file pone.0179595.s003.docx]

**Supporting information**

**S3 Table. Response evaluation in neuro-meningeal lymphomatosis.**

|  | GLOBAL | DLBCL | BL / Lymphoblastic Lymphoma | PCNSL |
| --- | --- | --- | --- | --- |
| Clinical CR | 17 (53%) | 8 (57%) | 4 (50%) | 4 (80%) |
| Clinical PR | 2 (6%) | 1 (7%) | 0 | 1 (20%) |
| Clinical SD / PD | 13 (41%) | 5 (36%) | 4 (50%) | 0 (0%) |
| CSF CR | 25 (78%) | 12 (86%) | 6 (75%) | 5 (100%) |

DLBCL: diffuse large B-cell lymphoma, BL: Burkitt lymphoma, PCNSL: primary central nervous system lymphoma, CR: complete response, PR: partial response, SD: stable disease, PD: progressive disease, CSF: cerebrospinal fluid.
